# Cognitive and balance dysfunctions due to the use of zolpidem in the elderly: a systematic review

**DOI:** 10.1590/1980-57642021dn15-030013

**Published:** 2021

**Authors:** Guilherme Tavares, Gizela Kelmann, Francisco Tustumi, Catherine Nardini Tundisi, Bárbara Regina Bruço Silveira, Bruno Maximiliano Augusto Colombo Barbosa, Diana Bragança Winther, Eduarda Conte Boutros, Gabriel dos Santos Villar, Giovanna Brunocilla, Gustavo Rodrigues Caldas Lourenção, Jiulia Giovanna Aranha Ferreira, Wanderley Marques Bernardo

**Affiliations:** 1 Department of Evidence-Based Medicine, Centro Universitário Lusíada – Santos, SP, Brazil.; 2 Department of Evidence-Based Medicine, Universidade de São Paulo – São Paulo, SP, Brazil.; 3 Department of Surgery, Hospital Israelita Albert Einstein – São Paulo, SP, Brazil.

**Keywords:** zolpidem, aged, postural balance, cognitive dysfunction, systematic review, zolpidem, idoso, equilíbrio postural, disfunção cognitiva, revisão sistemática

## Abstract

Zolpidem is one of the most widely prescribed hypnotic (non-benzodiazepine) agents for sleep disorder. Recently, an increase in the demand for this drug has been observed, mainly in the elderly population.

**Objective:**

This study aims to analyze the acute effect of zolpidem on cognitive and balance dysfunctions in the elderly population.

**Methods:**

A study was conducted by two independent researchers in four virtual scientific information bases and included randomized controlled trials. The studies evaluated elderly patients using zolpidem. Cognitive and balance dysfunctions were analyzed.

**Results:**

Six articles were included. The mean age of the participants in the studies was 69 years. The following zolpidem dosages were evaluated: 5, 6.25, 10, and 12.5 mg. Comparing zolpidem and placebo, relating to the cognitive dysfunctions, there is no statistically significant difference between the groups. However, in relation to balance dysfunctions, there is a statistically significant difference between the intervention and the comparison, favoring placebo.

**Conclusions:**

Zolpidem, even in usual doses (5 mg and 10 mg), has shown to increase the risk for balance dysfunctions. However, this does not occur in relation to cognitive changes.

## RESUMO.

Zolpidem é um dos agentes hipnóticos (não benzodiazepínicos) mais prescritos para o manejo dos distúrbios do sono. Recentemente, observou-se um aumento na demanda por esse medicamento, principalmente pela população idosa.

**Objetivo:**

Este estudo visa analisar o efeito agudo do zolpidem em relação às alterações cognitivas e de equilíbrio na população idosa.

**Métodos:**

Uma busca em quatro bases de informação científica virtual foi feita por dois pesquisadores independentes e incluiu ensaios clínicos randomizados. Os estudos avaliaram o uso de zolpidem em pacientes idosos. Alterações cognitivas e de equilíbrio foram analisadas.

**Resultados:**

Seis artigos foram incluídos. A média de idade entre os estudos foi de 69 anos. As seguintes posologias foram analisadas: 5; 6,25; 10; e 12,5 mg. Em relação às alterações cognitivas, comparando-se zolpidem com placebo, não há diferença estatisticamente significativa entre os grupos. Entretanto, no desfecho alterações de equilíbrio, há diferença estatisticamente significativa entre intervenção e comparação, a favor do placebo.

**Conclusões:**

Zolpidem, mesmo em doses usuais (5 e 10 mg), mostrou aumentar o risco para alterações de equilíbrio, entretanto, isso não ocorre em relação às alterações cognitivas.

## INTRODUCTION

Zolpidem is one of the most internationally prescribed hypnotic (non-benzodiazepine) agents, a derivative of imidazopyridine, and has a small action as an agonist at GABA-A receptors for treatment.^1^ It is used for sedation, anxiety reduction, and central muscle relaxation, and it has an anticonvulsant effect.^2^ In the elderly population, as they age, the consumption of hypnotics increases.^3^ Between 5 and 33% of the ageing population, from North America and the United Kingdom, received a prescription of benzodiazepine or a benzodiazepine receptor agonist for the sleep problem.^4^ ^,^ ^5^

Regarding balance dysfunctions, the z-drugs (non-benzodiazepine hypnotics) are associated with elderly hospitalization for fractures, injuries, and possibly falls.^6^ ^-^ ^8^ Besides that, cognition is critical for functional independence as people age, including whether someone can live independently, manage finances, take medications correctly, and drive safely. In addition, intact cognition is vital for humans to communicate effectively and for social life.^9^ Therefore, balance and cognitive dysfunctions have an important role in the quality of life of the elderly.

Currently, there are still controversies in the scientific literature about cognitive dysfunctions and the increased risk for falls due to zolpidem use in the elderly.^8^ ^,^ ^10^ ^,^ ^11^ We performed a systematic review of randomized controlled trials, aiming to analyze the acute effect of zolpidem on cognitive and balance dysfunctions in the elderly population.

## METHODS

This systematic review was submitted in the International Prospective Register of Systematic Reviews (PROSPERO) under trial registry CRD42020196434 and was conducted by the Department of Evidence-Based Medicine, Centro Universitário Lusíada, Santos, SP, Brazil.

The search for evidence was carried out independently by two researchers in the following virtual scientific information bases: Medline (PubMed), EMBASE, Central (Cochrane), Lilacs (VHL), and manual search, evaluating references from primary studies and reviews. The selection of studies was cross-checked and completed on March 4, 2021. The same search strategy was used in all scientific bases: (aged OR elderly OR older adult) AND (zolpidem OR non-benzodiazepine OR non-BZD).

The eligibility criteria were randomized clinical trials analyzing cognitive or balance dysfunctions as primary or secondary outcomes due to the acute effect of zolpidem usage in the elderly population (>60 years); no period or language limit was used; only the available full-text articles were included. The following study data were independently extracted by two researchers: name of the author and year of publication, study design, population studied, methods of intervention and comparison, the absolute number of events, means, deviation, standard error for the exams performed, and follow-up time. Data provided by the studies only in graphics were extracted using the WebPlotDigitizer software.^12^

Randomized clinical trials were assessed for risk of bias according to the following criteria: focal matter, randomization, blindfold allocation, double-blind, losses less than 20%, analysis by intention to treat, prognostic characteristics, outcome (importance, time, and method), sample calculation, and JADAD (scale ranging from 0 to 5 points, which takes into account randomization, double-blinding, and losses).

## RESULTS

**Figure 1.**
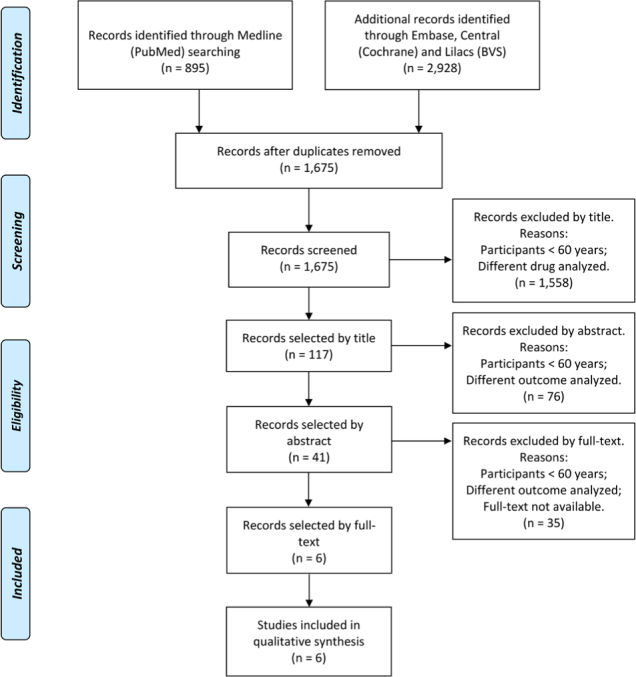
PRISMA flow diagram.

As shown in Figure 1, 895 articles were retrieved from Medline (PubMed) database and 2,928 articles from EMBASE, Central (Cochrane), and Lilacs databases. Excluding duplicates (2,148 studies), 1,675 articles remained to be selected; 117 articles were selected by title, 41 articles were selected by abstract, and finally, 6 articles were selected by full text.^13^ ^-^ ^18^ It was not possible to perform a meta-analysis considering the variety of analyzed outcomes, lack of data, and convergent measures. Qualitative analysis of this systematic review was then performed. The baseline characteristics of the included studies are given in Table 1. The risk of biases is given in Table 2.

**Table 1.** Baseline characteristics of the included studies.

Author and year of publication	Study design	Patients	Intervention	Control	Outcomes analyzed	Follow-up
Uemura et al., 2015^13^	Randomized controlled trial (RCT)/crossover	n=13 (2M and 11F) between 60 and 70 years, healthy, time to bed between 20 and 24 h.	Zolpidem (5 mg), Triazolam (0.125 mg), Rilmazafone (1 mg) — administered at bedtime — with 6 days of washout between treatments	Placebo — administered at bedtime — with 6 days of washout between treatments	CFF, SDR, SMT, body sway test, TUG and FRT	Outcomes analysis on the day after exposure, 6 days of washout — 4 periods
Boyle et al., 2009^14^	RCT/crossover	n=24 (7M and 17F), between 65 and 75 years, healthy, time to bed between 20 and 24 h.	Gaboxadol — 10 mg (n=24) and Zolpidem — 5 mg (n=23) — administered at bedtime — with 3 days of washout between treatments	Placebo (n=23) — administered at bedtime — with 3 days of washout between treatments	CFF, body sway test, and adverse events	Outcome analysis up to 12 h post exposure; 3 days of washout between treatment — 3 periods
Zammit et al., 2008^15^	RCT/crossover	n=11 (3M and 8F), between 65 and 81 years, healthy.	Zolpidem (10 mg) — n=11	Placebo — n=10	SOT and adverse events	Each treatment lasted 2 days, with a washout period of 2 days.
Hindmarch et al., 2006^16^	RCT/crossover	n=24 (10M and 14F), between 65 and 78 years	Zolpidem (6.25 mg) — n=23, Zolpidem (12.5 mg) — n=24 and Flurazepam (30 mg) — n=23	Placebo — n=23	CFF, CRT, CTT, DSST, memory recall, and adverse events	Each treatment lasted 2 days, with a washout period of 28–42 days. After 7–14 days post exposure, there was a medical evaluation.
Bentué-Ferrer et al., 2003^17^	RCT/crossover	n=49 (21M and 28F), between 64 and 78 years, healthy	Zolpidem (5 mg), Zopiclone (3.75 mg) and Lormetazepam (1 mg)	Placebo	CTT, LMT, Sternberg MRT, SRT, body sway test, and adverse events	Each treatment lasted 12 h, with a washout period of 8 days.
Fairweather et al., 1992^18^	RCT/crossover	n=24 (6M and 18F), between 63 and 80 years, healthy	Zolpidem (5 mg) and Zolpidem (10 mg)	Placebo	CTT, CRT, Sternberg MRT, and WRT	Each treatment lasted 7 days (3 treatments in total), with a washout period of 7 days. They were analyzed after the first day and the last.

TUG: The timed up and go; FRT: functional reach test; SDR: simple discrimination reaction; STM: short-term memory; CFF: critical flicker fusion; SOT: sensory organization test; CRT: choice reaction time; CTT: continuous tracking test; DSST: digit symbol substitution test; MRT: mean reaction time; LMT: learning memory task; SRT: simple reaction time; WRT: word recognition task; mg: milligram; M: male; F: female.

**Table 2.** Bias of included studies.

Author and year of publication	Focal matter	Randomization	Blindfold allocation	Double blind	Losses less<20%	Analysis by intention to treat	Prognostic characteristics	Outcome	Sample calculation	JADAD
Uemura et al., 2015^13^										2
Boyle et al., 2009^14^										4
Zammit et al., 2008^15^										3
Hindmarch et al., 2006^16^										4
Bentué-Ferrer et al., 2003^17^										3
Fairweather et al., 1992^18^										3

Green shade: absence of bias; red shade: presence of bias; yellow shade: doubt.

Considering all the studies, 145 patients were evaluated, 49 males and 68 females. The mean age of the participants in the studies was approximately 69 years. The mean washout period (crossover studies) between intervention and control (placebo) was approximately 10 days. The following zolpidem dosages were evaluated: 5, 6.25, 10, and 12.5 mg.

The results were divided into two main outcomes: (1) cognitive dysfunctions and (2) balance dysfunctions, as shown in Tables 3 and 4, respectively.

**Table 3.** Results: cognitive dysfunctions.

Author and year of publication	Intervention ´ control	Cognitive dysfunctions								
		CFF (Hz)	CTT	CRT
Uemura et al., 2015^13^	Zolpidem (5 mg) ´ placebo	I: 33 (SEM=0.19) C: 32.4 (SEM=0.18)	No data	No data
Boyle et al., 2009^14^	Zolpidem (5 mg) ´ placebo	Zolpidem minus placebo (MD) 1 h 30: -0.53 (-1.20 to 0.14) 4 h: -0.1 (-0.77 to 0.57) 8 h: -0.05 (-0.72 to 0.62)	No data	No data
Hindmarch et al., 2006^16^	Zolpidem (6.25 mg) and (12.5 mg) ´ placebo	Zolpidem minus placebo (MD) 6.25 mg: 0.2 (-0.38–0.77) 12.5 mg: -0.44 (-1.02–0.14) Performed 8 h after administration	Mean deviation (Pixels) (MD) 6.25 mg: -2.55 (-10.53–5.43) 12.5 mg: 4.20 (-3.78–12.18) Mean response time (ms) (MD) 6.25 mg: 19.59 (-113.56–74.39) 12.5 mg: 43.80 (-50.17–137.78)	Zolpidem minus placebo (DM) CRT - Recognition time (ms) 6.25 mg: -10.60 (-36.75 to 15.56) 12.5 mg: 11.18 (-14.97–37.33) CRT - Motor reaction time (ms) 6.25 mg: 9.16 (-12.51–30.82) 12.5 mg: 2.22 (-23.89–19.44) CRT - Total reaction time 6.25 mg: -1.44 (-35.13–32.26) 12.5 mg: 8.96 (-24.74–42.65)
Bentué-Ferrer et al., 2003^17^	Zolpidem (5 mg) ´ placebo	No data	Mean (±SEM) (mm) H0: I=11.41 (10.56–12.26) C=11.92 (11.13–12.71) H9: I=14.35 (12.44–16.26) C=12.32 (11.22–13.42)	No data
Fairweather et al., 1992^18^	Zolpidem (5 mg) and (10 mg) ´ placebo	No data	Placebo RMSTE (units) - Mean (SD): Day 1 – Baseline=11.4 (7.4); 10H=13.3 (12.4); 12H=14.0 (11.6); 18H=13.2 (11.2); Day 7 – Baseline=13.8 (14.4); 10H=14.0 (16.3); 12H=15.2 (22.0); 18H=13.7 (12.7). Peripheral stimuli (ms) - Mean (SD): Day 1 – Baseline=0.43 (0.08); 10H=0.45 (0.13); 12H=0.46 (0.08); 18H=0.46 (0.1); Day 7 – Baseline=0.45 (0.1); 10H=0.43 (0.08); 12H=0.44 (0.07); 18H=0.46 (0.11) Zolpidem (5 mg) RMSTE (units) - Mean (SD): Day 1 – Baseline=12.7 (10.4); 10H=12.8 (9.6); 12H=13.4 (9.9); 18H=13.5 (10.2); Day 7 – Baseline=11.7 (9.5); 10H=12.6 (9.6); 12H=12.1 (8.5); 18H=12.1 (10.1). Peripheral stimuli (ms) - Mean (SD): Day 1 – Baseline=0.43 (0.07); 10H=0.43 (0.1); 12H=0.46 (0.1); 18H=0.45 (0.6); Day 7 - Baseline=0.43 (0.07); 10H=0.44 (0.07); 12H=0.45 (0.07); 18H=0.45 (0.06). Zolpidem (10 mg) RMSTE (units) - Mean (SD): Day 1 – Baseline=12.5 (10.8); 10H=13.3 (9.9); 12H=13.2 (9.9); 18H=13.4 (11.9); Day 7 – Baseline=11.5 (8.1); 10H=12.4 (10.1); 12H=12.7 (11.3); 18H=13.5 (12.1). Peripheral stimuli (ms) - Mean (SD): Day 1 – Baseline=0.43 (0.08); 10H=0.44 (0.07); 12H=0.46 (0.08); 18H=0.45 (0.08); Day 7 – Baseline=0.44 (0.08); 10H=0.44 (0.08); 12H=0.45 (0.07); 18H=0.46 (0.09).	“There was no statistically significant difference.”
Uemura et al., 2015^13^	No data	Accuracy rate (%) I: 94.2 (SEM=1.1) C: 92.9 (SEM=0.9) Reaction time (s) I: 0.51 (0.01) C: 0.5 (0.01)	I: Accuracy rate (%)=34.6 (2.4) C: Accuracy rate (%)=32.1 (2.4)	No data	No data	No data	No data	No data
Boyle et al., 2009^14^	No data	No data	No data	No data	No data	No data	No data	No data
Hindmarch et al., 2006^16^	No data	No data	No data	Zolpidem minus placebo (MD) (nº) 6.25 mg: 0.68 (-1.24–2.61) 12.5 mg: 0.73 (-1.2–2.65)	No data	No data	No data	Zolpidem minus placebo (MD) (nº) Immediate word recall 6.25 mg: -0.34 (-1.47–0.78) 12.5 mg: -0.68 (-1.8–0.45) Delayed word recall 6.25 mg: -0.08 (-1.29–1.13) 12.5 mg: -1.05 (-2.26–0.16)
Bentué-Ferrer et al., 2003^17^	Mean (SEM) 2 digits H0: I=825 (42) C=985 (62) H9: I=892 (40) C=923 (56) 3 digits H0: I=926 (34) C=953 (45) H9: I=933 (33) C=950 (44) 4 digits H0: I=982 (39) C=995 (39) H9: I=950 (34) C=997 (41) 5 digits H0: I=1027 (39) C=1073 (50) H9: I=1010 (34) C=1039 (42) 6 digits H0: I=1123 (64) C=1136 (63) H9: I=1084 (41) C=1052 (36)	No data	No data	No data	IFRL (H9) (nº) I: 5 (mean) C: 4 (mean) MFIR (H9) (nº) I: 7 (mean) C: 7 (mean) DFR (H10) (nº) I: 6 (mean) C: 6 (mean)	Mean (±SEM) (ms) H0: I=344.97 (305.37–384.57) C=320.61 (308.21–333.01) H4: I=323.45 (315.05–331.85) C=338.29 (328.29–348.29) H5: I=335.13 (325.53–344.73) C=345.17 (333.17–357.17) H6: I=345.62 (333.62–357.62) C=347.65 (335.65–359.65) H8: I=336.50 (324.90–348.10) C=354.13 (340.53–367.73) H10: I=316.57 (310.17–322.97) C=331.00 (319.40–342.60)	No data	No data
Fairweather et al., 1992^18^	“There was no statistically significant difference.”	No data	No data	No data	No data	No data	“There was no statistically significant difference.”	No data

SDR test: simple discrimination reaction; STM test: short-term memory; CFF: critical flicker fusion; CRT: choice reaction time; CTT: continuous tracking test; DSST: digit symbol substitution test; MRT: mean reaction time; LMT: learning memory task; SRT: simple reaction time; WRT: word recognition task; SD: standard deviation; MD: mean difference; ms: meter per second; RMSTE: root mean square tracking error; s: seconds; IFRL: immediate free recall list; MFIR: mean of the four immediate recalls; DFR: delayed free recall; SEM: standard error of the mean; mg: milligram; mm: millimeter; p<0.05 in bold.

**Table 4.** Results: Balance dysfunctions.

Author and year of publication	Intervention ´ control	Balance dysfunctions			
		Body sway test	TUG	FRT	NeuroCom EquiTest SOT
Uemura et al., 2015^13^	Zolpidem (5 mg) ´ placebo	Mean (SEM) (cm) Eyes open I: 141.7 (1.7) C: 143.4 (1.7) Eyes closed I: 171.9 (2.3) C: 172.3 (2.3)	Mean (SEM) (s) I: 7.61 (0.05) C: 7.67 (0.05)	Mean (SEM) (cm) I: 311.8 (4.0) C: 288.4 (4.2)	No data
Boyle et al., 2009^14^	Zolpidem (5 mg) ´ placebo	Zolpidem minus placebo (GM) (cm^2^) Eyes open 1 h 30: 2.00 (1.62–2.46) 4 h: 1.78 (1.44–2.20) 8 h: 1.23 (1.00–1.52) Eyes closed 1 h 30: 1.65 (1.33–2.04) 4 h: 1.38 (1.11–1.71) 8 h: 1.09 (0.88–1.35)	No data	No data	No data
Zammit et al., 2008^15^	Zolpidem (10 mg) ´ placebo	No data	No data	No data	LS mean (SE) (%) ES1: -6.67±2.02 ES2: -7.41±1.90 ES3: -6.35±2.78 ES4: -13.14±3.76 ES5: -27.69±5.38 ES6: -17.21±3.61 CES: -15.83±2.88 Somatosensory ratio: -1.74±1.60 Visual ratio: -8.76±4.00 Vestibular ratio: -27.43±5.91 Preference ratio: 12.82±5.90
Bentué-Ferrer et al., 2003^17^	Zolpidem (5 mg) ´ placebo	Mean (±SEM) (mm^2^) Eyes open H0: I=194.04 (180.24–207.84) C=185.78 (173.86–197.7) H4: I=297.85 (274.84–320.86) C=218.73 (204.92–232.54) H5: I=332.63 (303.18–362.08) C=239.71 (222.22–257.2) H6: I=332.45 (308.52–356.38) C=275.41 (254.25–296.57) H8: I=301.90 (270.61–333.19) C=240.26 (222.77–257.75) H10: I=194.96 (183.00–206.92) C=188.54 (176.58–200.5) Eyes closed H0: I=452.27 (398.73–505.81) C=483.53 (436.68–530.38) H4: I=711.96 (605.99–817.93) C=454.30 (414.14–494.46) H5: I=613.57 (552.22–674.92) C=447.39 (416.15–478.63) H6: I=703.70 (634.54–772.86) C=533.06 (492.90–573.22) H8: I=637.67 (568.50–706.84) C=502.72 (455.87–549.57) H10: I=441.12 (398.73–483.51) C=456.76 (414.37–499.15)	No data	No data	No data

TUG: the timed up and go; FRT: functional reach test; ES: equilibrium score; CES: composite equilibrium score; SOT: sensory organization test; SEM: standard error of the mean; GM: geometric mean; cm: centimeter; mg: milligram; s: seconds; LS: least square; SE: standard error; p<0.05 in bold.

### Cognitive dysfunctions

#### Memory

##### Short-term memory

Four studies^13^ ^,^ ^16^ ^-^ ^18^ analyzed this outcome by carrying out five tests (see Table 3): Sternberg mean reaction time (MRT), word recognition task (WRT), short-term memory (STM), memory recall, and learning memory task (LMT).

The obtained data showed no statistically significant difference between zolpidem (5, 6.25, 10, and 12.5 mg) and placebo in relation to short-term memory.

##### Long-term memory

Two studies^16^ ^,^ ^17^ assessed this outcome by carrying out two tests (see Table 3): Memory recall and learning memory task (LMT).

The obtained results demonstrated no statistically significant difference between zolpidem (5, 6.25, and 12.5 mg) and placebo in relation to long-term memory.

#### Psychomotricity

Four studies^13^ ^,^ ^16^ ^-^ ^18^ analyzed this outcome by carrying out three tests (see Table 3): Continuous tracking test (CTT), choice reaction time (CRT), and simple discrimination reaction (SDR).

The obtained data demonstrated no statistically significant difference between zolpidem (5, 6.25, 10, and 12.5 mg) and placebo in relation to psychomotricity.

#### Vision

Four studies^13^ ^,^ ^14^ ^,^ ^16^ ^,^ ^17^ analyzed this outcome by carrying out two tests (see Table 3): Critical flicker fusion (CFF) and simple reaction time (SRT).

Uemura et al.^13^ performed the CFF and showed a statistically significant difference between zolpidem (5 mg) and placebo, in favor of zolpidem. Three studies^14^ ^,^ ^16^ ^,^ ^17^ demonstrated no statistically significant difference between zolpidem (5, 6.25, and 12.5 mg) and placebo in relation to vision changes.

#### Processing, attention, and concentration

One study^16^ assessed this outcome by carrying out the digit symbol substitution test (DSST) (see Table 3).

The obtained results showed no statistically significant difference between zolpidem (6.25 and 12.5 mg) and placebo in relation to processing, attention, and concentration.

### Balance dysfunctions

Four studies^13^ ^-^ ^15^ ^,^ ^17^ analyzed this outcome by carrying out four tests (see Table 4): Body sway test, the timed up and go (TUG), functional reach test (FRT), and NeuroCom EquiTest sensory organization test (SOT).

Uemura et al.^13^ performed two tests (body sway test and TUG) and showed no statistically significant difference between zolpidem (5 mg) and placebo in relation to balance dysfunctions. Regarding the FRT, the same study demonstrated a statistically significant difference between zolpidem (5 mg) and placebo, in favor of zolpidem.

Two studies^14^ ^,^ ^17^ carried out the body sway test and showed a statistically significant difference between zolpidem (5 mg) and placebo, in favor of placebo with eyes open and closed at 1 h and 30 min, 4 h, 5 h, 6 h, and 8 h after the zolpidem, and placebo administration.

Zammit et al.^15^ performed the NeuroCom EquiTest SOT and showed a statistically significant difference between zolpidem (10 mg) and placebo, in favor of placebo in conditions 1, 2, 4, 5, and 6, as well as in the balance score (CES) and in the vestibular rate.

## DISCUSSION

This study is a systematic review of randomized controlled trials assessing the acute impact of zolpidem on cognitive and balance dysfunctions in the elderly population. This study showed that the use of zolpidem in the elderly is not related to cognitive dysfunctions. However, zolpidem, even in the usual dosages (5 and 10 mg) may lead to balance dysfunctions.

The use of zolpidem in the elderly with sleep disorders provides better sleep quality, adding an independent and significant contribution to the quality of life beyond psychopathological symptoms.^19^ Besides that, the effects of sleep disorders are associated with a wide range of health conditions, including an increased risk of high blood pressure, diabetes, obesity, major depression, heart attack, and stroke.^20^ Thus, the aim of this study is to guarantee the quality of sleep and avoid such diseases without impairing cognition, whose integrity is of utmost importance to maintain communicative skills and, therefore, social life.^9^

This study showed no evidence in the scientific literature supporting any cognitive dysfunctions due to the use of zolpidem in the elderly population. However, recent randomized controlled trials evidenced a cognitive impairment relating to zolpidem in non-elderly.^21^ ^-^ ^23^ Stranks et al. performed a systematic review and showed that the performance on attention, verbal memory, and psychomotor speed were impaired compared to the control group, based on the data of middle-aged participants (mean age was 37 years) who ingested zolpidem before bedtime. Other cognition domains, such as speed of processing and working memory, were not affected.^24^

In addition to that, physicians should be aware of the greater susceptibility of the elderly to the negative consequences of zolpidem use and of other sedative hypnotics, especially the potential risk for falls, due to lower clearance rates and higher maximum serum concentration of these drugs.^25^ Fractures and injuries impair the functional capacity and the health-related quality of life in the long-term follow-up.^8^ ^,^ ^26^ Consequently, physicians should limit the treatment of these drugs when possible or use the lowest dose possible for patients at higher risk of imbalance. Chronic motor dysfunction of stroke patients^27^ and diabetic peripheral neuropathic patients^28^ present high individual risk of falling, and consequently, zolpidem should be used with caution in these patients. Also, osteoporotic patients are associated with an increased risk of related injuries,^29^ ^,^ ^30^ and zolpidem use should also be taken with precaution.

High-risk group patients should receive differentiated care, especially in activities at home, avoiding possible falls and encouraging certain physical exercises. de Kam et al.^31^ showed that exercise has positive effects on important predictors for falls and fractures, such as muscle strength, balance, and bone mineral density.

This study has some limitations. The included studies present a small number of patients, different doses of zolpidem, different treatment periods, and different analysis dates. Also, the studies used heterogeneous tests for the estimation of balance and cognition performances, which is why it was not possible to perform the meta-analysis.

The future of clinical research on this topic should stick to new randomized clinical trials, standardizing the tests for cognition and balance performed on the elderly population with a bigger sample size, allowing, then, a quantitative analysis of future systematic reviews. Future studies will allow us to consider the use of medication, by calculating the number needed to treat (NNT) or harm (NNH) to find out if the effectiveness in controlling insomnia and quality of life really outweighs the risks of cognitive and balance disorders.

The results of this study demonstrate that there is no statistically significant difference between zolpidem (5, 6.25, 10, and 12.5 mg) and placebo regarding cognitive dysfunctions (memory, psychomotricity, processing, attention, concentration, and vision). However, related to balance dysfunctions, the comparison between zolpidem (5 and 10 mg) and placebo showed that most articles present a statistically significant difference in favor of placebo, demonstrating that this drug produces relevant balance dysfunctions.
